# Overview and Evaluation of Chemicals and Methods for Flame Retardancy in Glued Laminated Wood Systems

**DOI:** 10.3390/polym17111459

**Published:** 2025-05-24

**Authors:** Ewelina Depczynska, Izabela Burawska

**Affiliations:** 1 Paged LabTech, Kwiatowa 1, 12-200 Pisz, Poland; 2 Department of Technology and Entrepreneurship in Wood Industry, Institute of Wood Sciences and Furniture, Warsaw University of Life Sciences—SGGW, 159 Nowoursynowska St., 02-776 Warsaw, Poland

**Keywords:** plywood, LVL, fire resistance, wooden construction, flame retardant

## Abstract

Due to the development of wooden construction as an ecological alternative to brick construction with a high carbon footprint, there is increasing interest in materials such as plywood and LVL (Laminated Veneer Lumber). These engineered wood products have many advantages compared to wood, such as a more uniform distribution of bending, shear, tensile, and compressive strength. However, they require improvements in fire and biological resistance. The flammability of wood and wood composites is a challenge that will allow these materials to stand out as structural or finishing materials. During combustion, toxic gases may be released, which can be harmful to people and the environment. Therefore, it is crucial to clarify whether fire-resistant wood materials are truly resistant to fire and non-toxic in fire conditions. On the other hand, flame retardants should not reduce the mechanical parameters of panels. This work analyses the current requirements (standards) regarding plywood intended for construction and the existing flame retardants for plywood and LVL based on the latest reports in the literature. We then propose an original method for evaluating future chemicals. Additionally, methods for assessing the flame retardancy of plywood and LVL based on the latest reports in the literature are described, and an original method for assessing flame retardancy methods is proposed.

## 1. Introduction

Plywood and LVL are valuable building materials that are used in construction due to the favorable strength parameters resulting from their layered structure [1]. Plywood is wood that is cut on the perimeter and glued in layers. It is considered to be the oldest composite material, as it was originally discovered by the Egyptians in 3600 BC. The most commonly used species of wood in plywood production are pine, birch, beech, alder, poplar, and exotic wood. Plywood, also known as “engineered wood”, is a natural material, which means it has a negative carbon footprint (CFP).

A characteristic feature of plywood is its high mechanical parameters, i.e., strength and stiffness. Thanks to its cross-shaped structure, it is possible to obtain the best properties with a low weight. Veneers with thicknesses of 0.35 mm to 3.3 mm are used for plywood production [2]. An LCA (life cycle assessment) was used to identify the environmental impact and key steps involved in plywood production [3].

**Figure 1:**
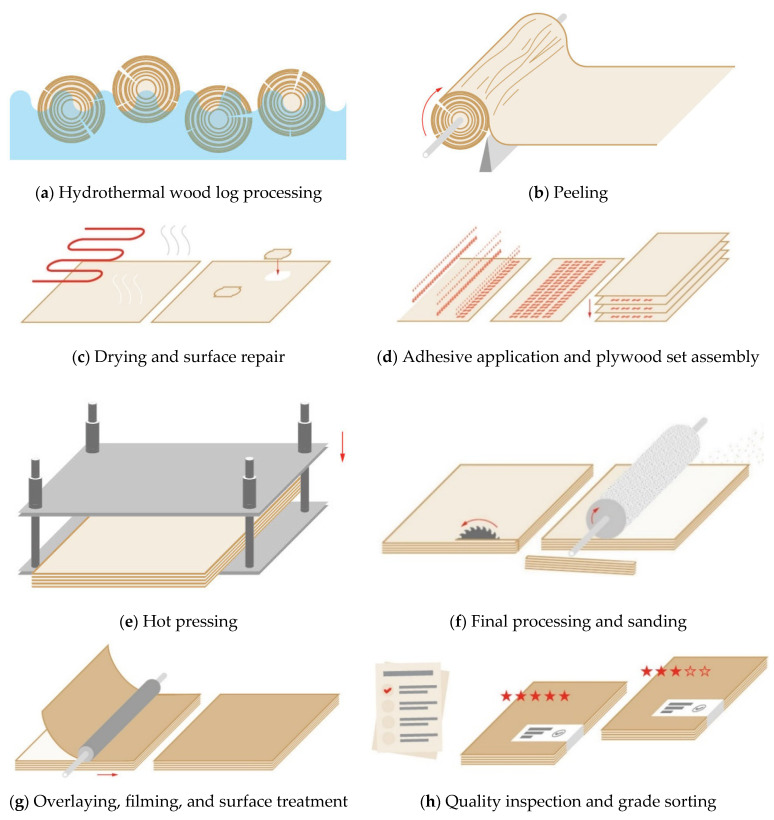
Plywood production process, according to https://sklejkapaged.pl/produkcja-sklejki/ (accessed on 20 May 2025).

Plywood production (shown in Figure 1) includes the following eight basic stages: (Figure 1a) hydrothermal wood log processing; (Figure 1b) peeling; (Figure 1c) drying and surface repair; (Figure 1d) adhesive application and plywood set assembly; (Figure 1e) hot pressing; (Figure 1f) final processing and sanding; (Figure 1g) overlaying, filming, and surface treatment; and (Figure 1h) quality inspection and grade sorting. Flame retardants can be added after plywood production (immersion impregnation) or before the formulation of sets (impregnation of veneers and re-drying). Plywood has a wide range of applications in construction. Examples of applications include formwork, prefabrication, scaffolding platforms, technical floors, roofs, modular homes, mobile homes, wall cladding, and stage platforms [2].

LVL is an important type of plywood used in structural applications, such as walls, roofs, and ceilings. The veneers used for LVL production are usually pine or spruce wood, with thicknesses of 2.5 to 5 mm in continuous production. There are two basic types of LVL: types P and C. LVL-P, where all veneers are oriented in one direction, is most often used for the construction of lintels, main beams, ridge beams, floor joists, roof rafters, trusses, frames, or beam reinforcement. In LVL-C, up to 20% of the veneers have a cross arrangement. It is used for roof, floor, and wall structures; prefabricated roof, floor, and wall elements and modules; and door panels [4].

Both plywood and LVL are combustible, with surface ignition at around 270 °C. According to Eurocode 5 [5], charring begins at a temperature of 300 °C [4]. Various chemicals are used to improve the fire resistance of plywood [2]. These chemical flame retardants for plywood and wood have been divided into the following four main groups: salt-based retardants, intumescent systems, chemical modification, and newer formulations [6].

### 1.1. Salt-Based Flame Retardants

Halide-based flame retardants, including chromium- and boron-containing compounds, are highly effective. Examples of such substances include magnesium chloride and ammonium chloride. Researchers have primarily focused on evaluating the impacts of these agents on plywood bonding quality and biological resistance, rather than assessing their toxicity. However, their Safety Data Sheets (SDSs) clearly indicate hazardous properties such as acute toxicity and eye irritation (e.g., ammonium chloride). Appropriate safety measures, including protective clothing, are essential during application. A major disadvantage of these solutions is the release of toxic and/or highly corrosive gases during combustion, which is harmful to both humans and the environment. Consequently, these substances are increasingly being phased out, especially in highly developed countries [7]. These compounds also negatively affect bending strength, particularly after prolonged exposure to stress [8].

Inorganic salts (such as hydroxides, carbonates, and sulphates) are considered environmentally friendly compounds. They exhibit good thermal properties; stability; and lower amounts of smoke and toxic, corrosive gases. However, their high water solubility (leaching) makes them unsuitable for outdoor use [9]. When used alone, these compounds are not sufficient as flame retardants, which is why they are often combined with substances such as urea (e.g., calcium carbonate with urea). It has also been found that melamine–urea–formaldehyde adhesives are more suitable for the production of fire-resistant plywood than urea–formaldehyde adhesives [10]. The most common and widely studied sulphate used in flame-retardant plywood is ammonium sulphate. Studies have shown a negative impact of this agent on the bonding quality of pine and alder plywood [11,12]. Combinations of biocidal agents (e.g., quaternary ammonium salts) with standard ammonium sulphate have also been tested, showing positive effects on both biological resistance and fire resistance [13]. However, ammonium sulphate also reduces bending strength [8].

Boron compounds, such as boric acid and borax, are effective flame retardants that offer high thermal and biological resistance, a neutral pH, and lower impacts on the mechanical properties of plywood compared to other retardants like phosphates. Studies have demonstrated that they have higher resistance to fungi responsible for white and brown decay, as well as termites, compared to phosphorus-based compounds such as ammonium monophosphate (MAP) and ammonium diphosphate (DAP) [14]. Another study identified a significant relationship between surface roughness parameters and the type of flame retardant used. The stylus method was applied to evaluate the surface properties of samples. Samples treated with a 3% borax solution had the smoothest surfaces, measuring 11.09 μm, while those treated with 6% boric acid had the roughest surfaces, measuring 12.44 μm. The results indicated that the surface quality of the panels declined as the concentration of the chemical treatment increased [1]. The effect of boric acid on plywood strength and adhesion has also been investigated. Fire-treated poplar plywood showed a 25.3% decrease in strength and a 64.9% decrease in its formaldehyde content compared to untreated panels [15]. Boron compounds can also be combined with phosphorus- or nitrogen-based flame retardants [7,16]. Additional advantages of boron-based chemicals include their non-toxicity, low cost, ease of application and processing, and long-term protection due to deep penetration. However, they are no longer used in the European Union due to concerns about potential health risks from prolonged exposure (use of personal protective equipment is recommended) [6].

Phosphorus-based flame retardants, such as phosphate esters, polyphosphonates, and phosphonic acid salts, are widely studied due to their high efficiency in improving plywood flame resistance and their environmental friendliness [17]. Phosphorus compounds can capture free radicals in the gas phase and promote char formation (carbonization) in the condensed phase, which inhibits heat transfer and the release of volatile pyrolytic products. Importantly, they do not produce toxic or corrosive gases during combustion. As a result, phosphorus-based flame retardants are commonly used in various polymers. Ammonium phosphate (APP) and guanyl urea phosphate (GUP) are well-established and effective flame retardants. During combustion, APP and GUP act as acidic and foaming agents, while wood provides the carbon source necessary for char layer formation [18]. In one study, beech and poplar veneers were immersed in solutions of ammonium monophosphate (MP) and sodium acetate (SA) using various impregnation times. Thermogravimetric (TG) and thermogravimetric derivatives (DTG) methods were used to assess the initial fire resistance of the veneers before manufacturing finished plywood. These analyses were performed on both treated and untreated wood samples, as well as on the flame retardants themselves. Additional properties of impregnated and un-impregnated veneers and plywood were evaluated, including solution uptake; weight gain from impregnation; the equilibrium moisture content; pH; and, for plywood specifically, mechanical strength and fire resistance.

Plywood fire resistance was assessed using a standard fire resistance test. The results showed that ammonium monophosphate was the most effective flame retardant—consistent with the results of the TG/DTG analyses—thus confirming the reliability of TG methods for predicting the fire resistance of final products [19]. The effects of flame retardants based on α-zirconium phosphate (α-ZrP) on plywood properties were also investigated. First, poplar veneers were treated with a cationic polyethyleneimine (PEI) polyelectrolyte resin and anionic polyelectrolyte ammonium polyphosphate (APP). Then, a urea–formaldehyde (UF) resin mixed with 9%, 15%, or 24% α-ZrP by weight was used to produce flame-retardant plywood. Two methods were applied: surface adsorption and adhesive blending. ZrP acted synergistically and catalytically, reducing the flammability of poplar plywood. The pHRR and THR values of the flame-retardant plywood were reduced by 41.8% and 22.9%, respectively. Furthermore, the presence of α-ZrP significantly reduced the concentration of toxic CO gas [20]. Phosphorus-containing compounds such as ammonium mono- and diphosphate are known to be effective flame retardants and have been used for many years in wood-based panels. However, studies have shown that they exhibit lower resistance to fungi that cause brown rot and white rot and termites compared to boron-based compounds. This limits their use primarily to indoor applications [9]. To improve their biological resistance, phosphorus compounds are often combined with quaternary ammonium salts [13].

Common nitrogen-based retardants include melamine and its derivatives, urea, and dicyandiamide. These compounds act by releasing nitrogen gas, which dilutes the flammable volatiles from wood, and are considered environmentally friendly. However, one disadvantage is the unpleasant odor produced during oxidation processes, such as the conversion of urea to ammonia [6]. Researchers have investigated the properties of plywood impregnated with a mixture of potassium carbonate and urea. A study evaluated changes in pH, wettability, color, and surface roughness. A melamine–urea–formaldehyde resin was used as the adhesive, and properties such as shear strength and formaldehyde emission were assessed. It was found that impregnated plywood exhibited increased strength, pH, and color change, without affecting surface roughness. However, impregnation negatively impacted the adhesion quality of the plywood, though it also reduced the formaldehyde emissions from the final product. It can be concluded that a melamine–urea–formaldehyde adhesive is more suitable for the production of fire-resistant plywood than a urea–formaldehyde adhesive [10]. Similar to phosphorus-based compounds, nitrogen-based flame retardants tend to reduce the bonding quality (shear strength) of plywood [8,12].

Examples of combined phosphorus- and nitrogen-based flame retardants include ammonium mono- and diphosphate, ammonium polyphosphate, polyols, amides, water–organic dispersions, metal oxides and organic additives, cyclic phosphazene derivatives, aziridinylphosphazene, and polymers of ammonium phosphate and guanylurea phosphate. The use of these compounds in mixtures is justified by their differing mechanisms of action. Nitrogen compounds work in the gas phase by diluting volatile wood compounds and reducing oxygen levels, while phosphorus compounds promote charring on the surface [6]. One example of this type of application is a mixture of guanylurea phosphate, boric acid, and ammonium phosphate used to apply a flame-retardant treatment to plywood in an autoclave. Results showed that continuous exposure to temperatures up to 65 °C over the course of one year had minimal impact on the strength properties of plywood. The types of chemicals used had a greater influence on mechanical properties than the duration of exposure. The modulus of elasticity (MOE) and modulus of rupture (MOR) values remained relatively stable over the exposure period, though treated material showed a greater tendency toward degradation than untreated material [16]. While such combined systems offer synergistic, positive effects on flame retardancy, they also share the disadvantages of both components, particularly the reduced bonding quality of plywood. In one study, six commercially available flame retardants and three laboratory-prepared phosphorus- and nitrogen-based agents were analyzed. The results showed that the addition of flame retardants progressively reduced the bond strength of plywood as the molar ratio of the formaldehyde in the resin increased. Additionally, the impact of the flame retardants on the curing time of the UF resin decreased as the formaldehyde content increased. The rate of formaldehyde emission varied depending on the formaldehyde content in the flame retardant [15]. Silicone and organic silicate systems are also characterized by high thermal resistance, making them promising candidates for fire-resistant coatings and other fireproof solutions [14,21]. These materials can be incorporated into adhesives. It has been proven that silicone-based adhesives improve the fire resistance of plywood compared to a PU (polyurethane) adhesive. However, maintaining bonding quality remains a challenge. Silicone adhesives positively affect two key aspects of fire performance: fire resistance (resistance to high temperatures) and reaction to fire (the rate of spread in a structure) [22].

Examples of nanocomposite flame retardants include vermiculite and carbon nanotubes. Due to their highly developed surface areas, these materials have potential as flame retardants. However, they have not yet demonstrated satisfactory performance as standalone solutions [6].

### 1.2. Intumescent Fireproofing Systems

These systems are non-toxic, aesthetically pleasing solutions that can delay the spread of fire within a building. When exposed to high temperatures, they swell and expand their volumes by 50 to 200 times, forming thick, porous, charred layers that act as protective barriers, providing resistance to both heat and mass transfer. They are lightweight and can be applied in relatively thin layers, enabling a thermal insulation effect for a specified duration. The swelling process is a combination of charring and foaming at the interface between the gas and the condensed phase. The char layer formed by the intumescent coating is easily damaged at high temperatures, and the coating itself is susceptible to aging. Additionally, most water-based fireproof coatings exhibit poor adhesion to substrates, which leads to delamination at elevated temperatures, significantly reducing their fire-protection capabilities. These systems vary considerably in quality, which depends on their formulation. Application methods are also limited and are typically restricted to rollers and brushes [6].

Researchers have developed dual-function, intumescent, fire-retardant/self-healing coatings for plywood protection. These coatings contain ammonium polyphosphate (APP) as a source of acid and gas, polyvinyl alcohol (PVA) as a source of carbon and a self-healing agent, and sodium silicate and sodium fluorosilicate (SS) as precursors for forming inorganic networks. The resulting semi-transparent coatings inhibit fire development through an interaction between the inorganic salts (silicates) and the intumescent flame-retardant system. The self-healing property of the coating is achieved through hydrogen bonding in the PVA. These semi-transparent coatings exhibit both fire resistance and self-healing capabilities. Studies confirmed the potential use of PVA/SSA coatings for protecting plywood intended for construction applications [23].

Studies show that the main components of intumescent coatings can include a mixture of urea–formaldehyde resins and polyvinyl acetate. Additionally, a tested intumescent fire-retardant system consisted of guanylurea phosphate (GUP), ammonium polyphosphate (APP), pentaerythritol (PER), and melamine (MEL). Plywood samples coated with a commercial intumescent paint, the synthesized coating, and individual main coating components, as well as uncoated plywood, were analyzed using a cone calorimeter. Overall, the fire- and smoke-resistance properties of the synthesized coating were significantly better than those of the commercial product [24]. Acrylic binders have also been used to formulate intumescent coatings. In one study, plywood was protected with an intumescent system composed of an acrylic emulsion as a binder, pentaerythritol as a carbon source, melamine as a foaming agent, and ammonium polyphosphate as a dehydrating agent. Fire performance tests conducted with a cone calorimeter showed that this system significantly improved the fire resistance of the plywood [25].

Intumescent materials such as acrylic copolymers are also used to enhance the fire resistance of wooden materials in indoor applications. In another study, various acrylic copolymers were mixed with different amounts of organic clays (1, 3, 5, and 10%). The results demonstrated that the intumescent formulations significantly improved the fire resistance of plywood and effectively delayed the spread of fire [26].

### 1.3. Chemical Modification of Plywood

Acetylation of wood is a process that uses acetic anhydride. It has been proven effective in increasing the hydrophobicity of wood, reducing dimensional changes, protecting against biological degradation, extending ignition time, and reducing both fire reactions and smoke production. Studies have been conducted on beech plywood and ash wood [6].

Modifying beech plywood with a phenol–melamine–formaldehyde resin and DMDHEU (Figure 1a) is a highly effective process due to the deep penetration of these substances and their permanent reactions with wood components. As a result of a condensation reaction between the methyl groups in the resin and esterification between the methyl and hydroxyl groups in softwood (e.g., pine), a three-dimensional rigid network is formed. These systems exhibit high fire resistance and are additionally characterized by low toxicity and reduced smoke generation [6].

### 1.4. Other Chemicals

**Figure 2:**
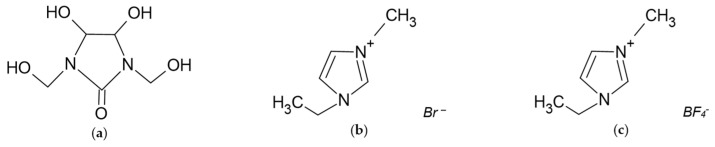
Chemical structures of (**a**) DMDHEU, (**b**) 1-ethyl-3-methylimidazolium bromide, and (**c**) 1-ethyl-3-methylimidazolium tetrafluoroborate.

Examples of ionic liquids used for flame retardancy include 1-ethyl-3-methylimidazolium bromide (Figure 2b) and 1-ethyl-3-methylimidazolium tetrafluoroborate (Figure 2c). These substances offer several advantages, such as melting points close to ambient temperature, low viscosity, high solubility, low volatility, non-flammability, and biological resistance. However, their disadvantages include a higher cost compared to currently used solutions and a relatively limited number of reports in the literature [6].

Expanded graphite appears to be a promising flame-retardant material when used either independently or as an additive in intumescent paints. It can counteract the primary cause of thermal degradation of cellulose (the main component of wood), namely its crystallization temperature. The drawbacks of expanded graphite include its sulfur content, which is environmentally unfavorable, and its dark color [27].

Pyrolysis (surface charring) is not widely used on an industrial scale due to the need for direct flame application. While this method results in a hydrophobic surface, it has notable disadvantages, including a blackened appearance and only partial effectiveness [6]. A supramolecular flame retardant for plywood was developed through vacuum impregnation and high-temperature in situ polymerization. The study, conducted on poplar wood, involved a prepolymer composed of phytic acid, urea, and dicyandiamide, which polymerized with chitosan or lignocellulose to form a supramolecular flame retardant. This agent reduces flammability and prevents mass loss via in situ polymerization [15].

The significance of plywood continues to grow in the construction and furniture industries due to its favorable mechanical properties [12]. As the use of plywood in construction expands, researchers are actively working to enhance its fire resistance [9]. During combustion, toxic gases may be released, posing risks to both human health and the environment. Therefore, it is essential to ensure that fire-resistant, wood-based construction materials are not only resistant to fire but also do not release harmful gases in the event of a fire [18]. The mechanical properties of flame-retardant plywood are influenced not only by the thickness of the veneers used but also by processing parameters such as the pressure, the temperature, the type of adhesive, and the quantity applied. Researchers evaluated the physical and mechanical properties of plywood made from birch and alder veneers. Tests were conducted on shear strength, bending strength, and compressive strength for plywood produced from both compressed and uncompressed veneers [28]. The results indicated that the overall mechanical performance of the veneers and plywood improved with an increase in the veneer compression ratio from 5% to 15%. This also led to reductions in adhesive consumption of up to 20%, as well as a 40% improvement in the surface properties of the veneer samples. Plywood samples required lower pressures ranging from 25% to 30% when manufactured from regular (uncompressed) veneers. Therefore, altering the veneer compression process can be considered a promising alternative method for enhancing the physical and mechanical properties of experimental plywood panels intended for use in construction [28].

### 1.5. Mechanisms of Bonding Flame Retardants to Wood, Flame-Retardant Mechanisms, and Their Environmental Aspects

Different types of flame retardants exhibit various mechanisms in their interactions with wood, which affect their effectiveness, durability, and application methods. The main types of bonding resulting from interactions between flame retardants and wood are as follows:

1. Physical adsorption—e.g., inorganic salts (borates, sulfates, and phosphates) deposit on the wood surface without forming permanent chemical bonds [29].
2. Chemical (covalent) bonding—organic phosphates or silanes react with hydroxyl groups in cellulose/lignin, forming stable covalent bonds [30].
3. Penetration and cross-linking—some polymeric flame retardants (e.g., APP) penetrate into wood cells and undergo cross-linking or polymerization processes, enhancing the durability of their protection [15,31].
4. Surface barrier—coating-based retardants form a physical protective layer without deep interaction, limiting oxygen access and ignition sources [32,33].

During their service lives, timber elements treated with fire retardants can be exposed to varying degrees of environmental impacts [34]. Among the weathering factors that influence the properties of flame retardant-treated wood, the most significant are solar radiation (UV, IR, and visible light), which can cause hydrolysis of wood components; temperature, which may induce photochemical degradation; and moisture, which can lead to leaching of active ingredients [34]. The durability and weathering resistance of flame retardants in combination with wood have been widely studied by numerous research groups. Most publications focus on changes in fire resistance, while only a few consider changes in appearance and aesthetic properties, which are often just as important to investors as fire-protection performance. The deterioration of properties of treated wood may result from two main mechanisms, as described by Östman and Tsantaridis [35]. The first mechanism involves the migration of fire-retardant substances within the wood and their crystallization on its surface. This process typically occurs when both a high wood moisture content and high relative humidity are present. The second mechanism is associated with the leaching of a fire retardant from the wood volume, leading to a reduced amount or an altered composition of the active components in the treated element. Additionally, the durability of fire-retardant coatings can vary significantly depending on the wood species to which they are applied [36].

The long-term performance of the primary groups of wood fire retardants (phosphorus-, boron-, and nitrogen-based compounds) is a key aspect of fire-protection efficiency, especially under environmental aging (time and exposure to humidity, UV radiation, and other weathering factors). Phosphorus-based flame retardants decompose under heat to form phosphoric acids or their derivatives, which catalyze the dehydration of wood components [33]. This leads to the formation of a compact, carbonaceous char layer that serves as a thermal insulator. In terms of chemical durability, phosphorus compounds are moderately resistant to leaching but are not photostable and can degrade under UV exposure. The environmental effects after combustion of phosphorus-based fire retardants are generally favorable. These retardants are widely used due to their low toxicity, minimal release of harmful gases, and non-corrosive residues under proper combustion conditions. These compounds do not produce acutely toxic or ozone-depleting emissions, making them a safer choice in fire scenarios [37].

Boron compounds act by suppressing combustion reactions and reducing the amount of heat released during burning [38]. However, they are highly hygroscopic and thus extremely susceptible to leaching. Their effectiveness and durability are low unless stabilized with hydrophobic or silicon-based additives. The environmental effects after combustion of boron-based flame retardants are generally considered low-risk. Upon exposure to fire, these compounds decompose or melt, often forming glassy boron oxide (B_2_O_3_). They are clean-burning and environmentally preferable, as no toxic gases are generated [37]. Nitrogen compounds decompose thermally, releasing gases such as nitrogen (N_2_) or ammonia (NH_3_), which displace oxygen near the wood surface [39]. These gases also cool the combustion zone through endothermic reactions and reduce the concentration of flammable volatile products released from the wood. Some nitrogen compounds also form protective foams that insulate the substrate from flames. However, their performance declines with water exposure due to leaching or chemical degradation. The environmental effects after combustion of nitrogen-based fire retardants are generally not severe, but they are not entirely benign either. Common nitrogen-based retardants (e.g., melamine, urea, and dicyandiamide) release nitrogen-containing gases during decomposition, which may impact air quality and leave reactive residues [40].

The issue of the durability of the reaction to fire is particularly important in the case of elements such as wall claddings/facades, which are directly exposed to weather conditions. Therefore, flame retardants should be selected in a thoughtful manner and strictly adapted to future applications [41].

The aim of this work was to review and assess existing methods for producing flame-retardant plywood in accordance with the current state of technology. The subject of this work was flame-retardant plywood made of various types of wood, with thicknesses ranging from 9 to 40 mm, intended for both external and internal construction applications: walls, floors, and ceilings. Additionally, flame-retardant agents were analyzed, and based on this analysis, an original method for evaluating the effectiveness of the agents was proposed.

## 2. Main Plywood Testing Standards Intended for Construction

Increasing requirements for the fire-resistant properties of wooden structures and elements to meet new standards have been an important part of building regulations over the last decade.

According to the EN 13986:2004+A1:2015 standard [42], wood-based panels used in construction must have appropriate properties determined by appropriate testing methods. Based on the minimal guaranteed parameters obtained, panels are divided into the following categories:

- For internal use as structural elements in dry conditions;
- For internal use (or protected externally) as structural elements in humid conditions;
- For external use as structural elements;
- For internal use as non-structural elements in dry conditions;
- For internal (or protected external) applications as non-structural elements in humid conditions;
- For external use as non-structural elements;
- For use as structural floor coverings on joists in dry or humid conditions or externally;
- For use as structural roof coverings on joists in dry or humid conditions or externally;
- For use as structural wall coverings on studs in dry or humid conditions or externally.

**Figure 3:**
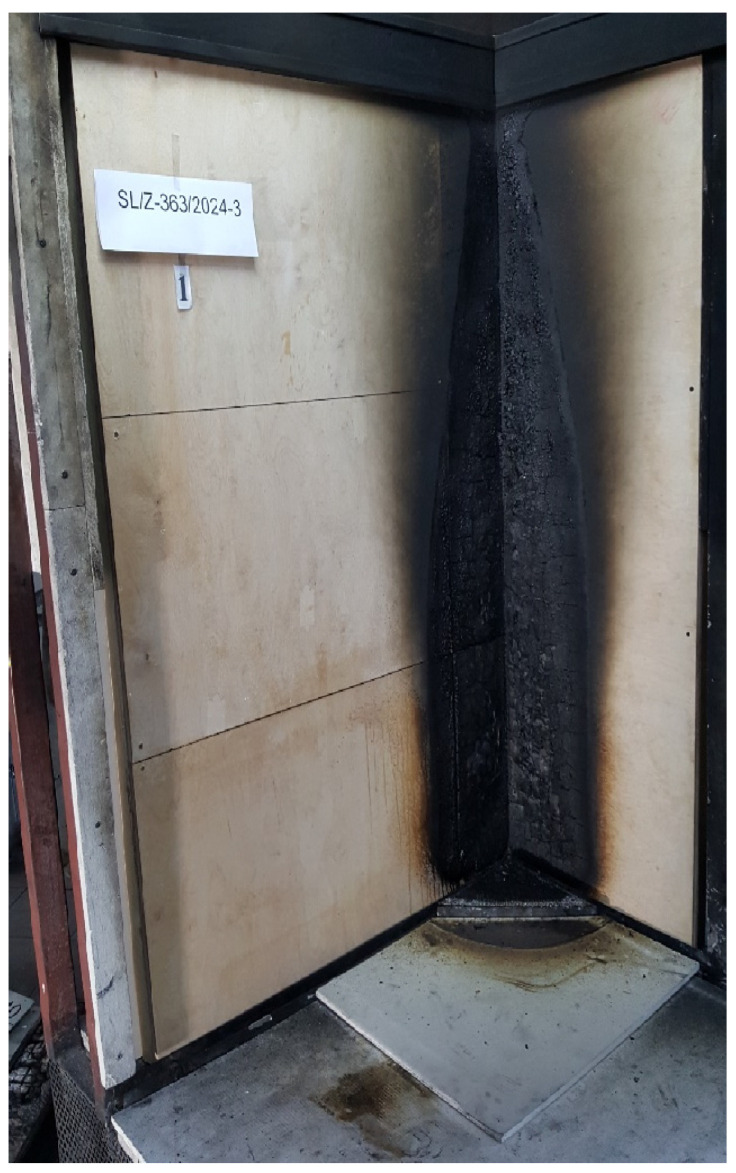
SBI (Single Burning Item) test conducted according to [46].

**Figure 4:**
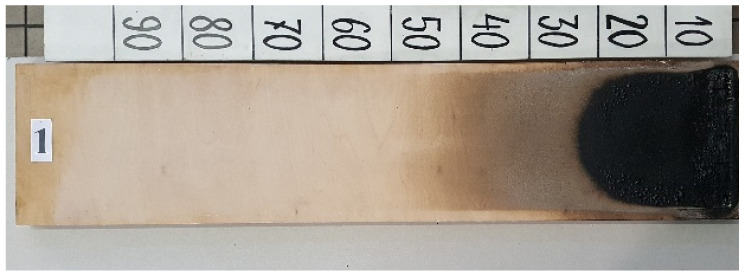
Floor testing in accordance with [48].

The above standard details the conformity assessment and the requirements for marking these products. The document covers wood-based panels in the form of solid wood panels, LVL, plywood, OSB panels, resin- or cement-bonded particleboards, wet-processed fiberboards (hard-, medium-, and softboards), and dry-processed fiberboards (MDF) for use in construction. The above panels may contain chemicals that improve their reaction to fire and resistance to biological attacks, such as from fungi and insects. The standard also refers to other standards that plywood should fulfil, such as EN 636:2005 [43] and EN 314-2:2001 [44]. According to the above, the required minimum fire reaction class for plywood in accordance with EN 13501-1:2019-02 [45] is Ds2d0, where D means that the flammability with a small flame for 60 s meets the criterion Fs < 150 mm, indicating significant participation in fire; s2 indicates medium smoke emission; and d0 indicates there are no burning droplets. However, requirements can be individual, and very often investors require class Bs1d0 in the case of walls and ceilings or Bfls1 in the case of floors, where B means that the flammability with a small flame for 60 s meets the criterion Fs < 150 mm, indicating limited participation in fire. The above markings are made in accordance with the standards EN 13823+A1:2014-12 [46], EN ISO 9239-1:2004 [47], and EN ISO 11925-2:2020-09 [48]. During testing, parameters such as FIGRA [W s-1] (a parameter describing the rate of heat release by the tested material), THR [MJ] (a parameter describing the total heat released), and SMOGRA [m^2^ s^−2^] (the smoke release rate index) are determined. Figure 3 and Figure 4 present samples tested in accordance with the above standards.

The flammability of plywood and LVL is also included in PN-EN 1995–1-2:2008 Eurocode 5 [16]. The difference is that the Eurocode focuses on REI values for load-bearing and separating structures. These requirements are expressed in minutes and vary depending on the intended application of the plywood within a building (e.g., walls, floors, roofs, and load-bearing elements). The specific requirements are presented in Table 1. Important standards for evaluating the mechanical properties of plywood for construction purposes include PN-EN 789:2005 [49] and EN 310:1994 [50].

Another key document for manufacturers is regulation (EU) No. 305/2011 of the European Parliament and of the Council of 9 March 2011 [52], which established conditions for the marketing of construction products and repealed Council Directive 89/106/EEC (CPR regulation). This regulation imposes several obligations on manufacturers, including the need for a European technical assessment and the preparation of a DoP (Declaration of Performance). Fire resistance class requirements also depend on the height of the building. These requirements are detailed in Table 2.

## 3. Methods to Increase Flame Retardancy of Plywood

Surface impregnation (non-pressure) is a method for protecting wood to a depth of up to 2 mm. It is mainly used for applications involving intumescent paints and varnishes [6,24,26,27]. The disadvantages of this method include being time-consuming and providing only superficial protection of the plywood. The most common surface impregnation techniques include pneumatic spraying, hydrodynamic spraying, and the use of rollers or brushes [28].

Pressureless baths are a procedure in which cold plywood (at room temperature) is immersed in a hot bath (60–80 °C) or hot plywood (60–80 °C) is immersed in a bath at room temperature. This process is commonly referred to as a hot–cold bath. The process lasts up to several hours, depending on whether it is performed on wet or dried veneers. It has been proven that a high veneer moisture content positively influences the diffusion process during impregnation and can significantly reduce the required treatment time [17]. Both entire plywood panels and individual veneers can be impregnated. However, when treating whole plywood panels, the process may be less effective due to the presence of adhesive layers between veneers, which can act as barriers. Impregnating individual veneers prior to plywood assembly is more effective. Nevertheless, uneven application of the impregnating agent, caused by variations in the wood structure and absorptivity, can negatively affect bonding quality and the overall integrity of the finished plywood [19,53].

Pressure impregnation in an autoclave is a highly effective method that can last up to 2 h. This process is usually carried out in three stages. In the initial vacuum stage, air is extracted from the wood [15]. The main impregnation takes place under high pressure, typically between 6 and 8 bar. The final stage involves removing excess flame retardant that was not absorbed by the wood. After this process, the veneers or entire plywood panels are dried again. Producing plywood from pre-impregnated veneers ensures flame retardancy throughout the entire cross-section of the material [10]. The advantage of this method is deep and highly uniform impregnation (penetration depths exceeding 8 mm). The main disadvantage, when impregnating whole plywood panels, is a reduction in adhesive quality, specifically lower shear strength compared to untreated plywood. This technique is compatible with all flame retardants based on phosphorus, nitrogen, or boron [6,19].

## 4. Original System for Solution Evaluation

After analyzing existing flame retardancy methods and drawing on our own experience, an original system for assessing the effectiveness of solutions was proposed. Five key criteria were identified as the most relevant for effective evaluation: the penetration depth, uniformity of application, processing time, impact of the process on the mechanical parameters of plywood, and cost. A simple three-point rating scale was developed. For each parameter, specific ranges or qualitative guidelines were defined.

In the case of two criteria (the uniformity of application and impact on mechanical properties), the assessment is more general, relying on empirical knowledge and findings from multiple publications. Unfortunately, research methodologies in the literature vary significantly, and results often depend on the agent used and the specific conditions under which the material was tested. The scale is presented in Table 3.

The basis for the evaluation of plywood flame retardancy methods was an analysis of the available professional literature and our own experience with the use of various plywood flame retardancy methods. The results of the analysis are presented in Table 4.

## 5. Original System for Evaluating Flame Retardants

Based on the analysis of flame-retardant agents, an original method of assessing the effectiveness of the agents was proposed. Ten key criteria were identified for evaluating their performance. Similar to the assessment of the flame retardancy methods, a three-point rating scale was used. Specific ranges were established for each parameter, depending on its nature. However, the evaluation of flame-retardant agents was complicated by several factors. Even within a single group—such as phosphates—the number of available chemicals was extensive. Further challenges arose from the use of varying research methodologies, incomplete or inconsistent test data, and the frequent application of mixtures or differing concentrations, as well as differences in wood species and impregnation techniques.

As a result, efforts were made to provide the most accurate yet general assessment possible. Consequently, many parameters were evaluated descriptively. Despite these limitations, the proposed method allowed for a sufficiently general and practical evaluation of flame-retardant chemicals. The rating scale is presented in Table 5. The results of the analysis of selected flame retardants are shown in Table 6.

## 6. Summary

The conducted point analysis indicates that the most effective method for producing flame-retardant plywood is pressure impregnation of individual veneers. If an autoclave is not available, the immersion method is a viable alternative. Among the surface application methods, spray techniques (pneumatic or hydrodynamic) proved to be the most effective. Naturally, the final choice of application method will also depend on the properties of the flame-retardant agent used.

There is no universal application method suitable for all flame retardants, primarily due to the differing chemical natures of these compounds and variations in their rheological properties, such as viscosity and density.

The analysis shows that the most effective flame retardants (with the highest scores) are ionic liquids. Unfortunately, they are not yet widely used due to their high price. Chemical modifications of wood are also very effective. While the most commonly used commercial products are based on phosphorus, nitrogen, or boron, they are not the most effective options available. Nonetheless, they remain widely used due to their low cost and relatively high efficiency.

## Figures and Tables

**Table 1:** Fire resistance classes of buildings [51].

Fire Resistance Class of Building	Main Supporting Structure	Roof Structure	Roof	External Wall	Internal Wall	Roof Covering
A	R240	R30	REI120	EI120	EI60	EI30
B	R120	R30	REI60	EI60	EI30	EI30
C	R60	R15	REI60	EI30	EI15	EI15
D	R30	-	REI30	EI30	-	-
E	-	-	-	-	-	-

**Table 2:** The fire resistance class requirements depending on the building height [52].

Maximum Fire Load Density in Fire Zones (Q) [MJ/m^2^]	Single-Storey Building	Multi-Storey Building			
		Short	Medium High	Tall	High-Rise
Q ≤ 500	E	D	C	B	B
500 < Q ≤ 1000	D	D	C	B	B
1000 < Q ≤ 2000	C	C	C	B	B
2000 < Q ≤ 4000	B	B	B	None	None
Q > 4000	A	A	A	None	None

**Table 3:** Scale for assessing impregnation methods.

Assessment Criteria	Scoring		
	1	2	3
Penetration depth	up to 2 mm	2–8 mm	above 8 mm
Quality of final surface	low	medium	high
Processing time for 100 m^2^	above 8 h	2–8 h	less than 2 h
The influence of the process on mechanical parameters	high	medium	low or none
Cost for 100 m^2^	above EUR 250	EUR 25–EUR 250	less than EUR 25

**Table 4:** Evaluation of impregnation methods.

Assessment Criteria	Surface Impregnation (Pressureless)						Pressureless Impregnation (Immersion)		Pressure Impregnation (Autoclave)	
	Pneumatic Spray	Airless Spray	Roller	Brush	Rollers/Layers	Lubrication	Whole Plywood	Single Veneers	Whole Plywood	Single Veneers
Penetration depth	1	1	1	1	1	1	2	3	2	3
Quality of final surface	3	3	1	1	2	1	1	2	2	3
Processing time for 100 m^2^	2	2	1	1	2	1	3	3	3	3
The influence of the process on mechanical parameters	3	3	3	3	3	3	2	1	1	2
Cost for 100 m^2^	3	3	2	2	3	2	3	3	3	3
Sum	12	12	8	8	11	8	11	12	11	14

**Table 5:** Assessment scale for impregnating agents.

Assessment Criteria	Scoring		
	1	2	3
FIGRA effectiveness	low	medium	high
THR effectiveness	low	medium	high
Harmful to the environment	high	medium	low
Harmful to humans	high	medium	low
Application	interior	internal and external under roof	exterior
Impact on mechanical parameters	high	medium	low or none
Visual (decorative) aspects	negative	medium or none	positive
Durability—resistance to fungi	low	medium	high
Durability—water resistance	low	medium	high
Cost for 100 m^2^	less than EUR 250	EUR 250–EUR 1250	above EUR 1250

**Table 6:** Evaluation of impregnating agents.

Assessment Criteria	Halides	Inorganic Salts	Boron Compounds	Phosphorus Chemicals	Nitrogen Chemicals	Mixtures of Phosphorus and Nitrogen Chemicals	Silicon-Based Compounds	Nanocomposites	Intumescent Fireproofing Systems	PF-Me and DMDHEU	Acetylation	Pyrolysis	Ionic Liquids
FIGRA	2	1	3	1	1	2	1	1	3	3	3	3	3
THR effectiveness	3	1	3	3	3	3	1	1	1	1	3	1	3
Harmful to the environment	1	2	2	3	3	3	3	1	3	3	3	3	3
Harmful to humans	1	2	1	3	3	3	3	1	3	3	3	3	3
Application	1	3	2	1	2	2	3	2	2	3	3	2	3
Impact on mechanical parameters	2	2	1	2	1	1	1	1	3	2	1	1	3
Visual (decorative) aspects	1	2	2	1	1	1	2	1	1	1	1	1	3
Durability—resistance to fungi	3	2	3	1	3	2	3	3	2	3	3	1	3
Durability—water resistance	1	1	2	1	1	1	3	3	1	2	3	1	2
Cost for 100 m^2^	3	3	3	3	3	3	2	1	1	2	3	3	1
Sum	18	19	22	19	21	21	22	15	20	23	26	19	27

## Data Availability

Not applicable.

## Abbreviations

The following abbreviations are used in this manuscript:

DoP	Declaration of Performance;
CFP	negative carbon footprint;
SBI	Single Burning Item;
FIGRA	a parameter describing the rate of heat release by the tested material;
SMOGRA	the smoke release rate index.
